# Hydrocodone-Induced Delirium in an Otherwise Healthy 20-Year-Old Male

**DOI:** 10.31486/toj.24.0129

**Published:** 2025

**Authors:** Andrew Ginsberg, Ravi Chauhan

**Affiliations:** ^1^ Department of Emergency Medicine, Ochsner Clinic Foundation, New Orleans, LA; ^2^ The University of Queensland Medical School, Ochsner Clinical School, New Orleans, LA

**Keywords:** *Adverse effects*, *analgesics*, *analgesics–opioid*, *drug-related side effects and adverse reactions*, *risk factors*

## Abstract

**Background:**

Hydrocodone is a commonly prescribed analgesic for acute, chronic, and postoperative pain because of its relatively weaker strength compared to other opioids and its generally effective symptomatic control. Like any medication, however, hydrocodone is associated with side effects. An infrequent side effect is delirium.

**Case Report:**

A 20-year-old male with no psychiatric medical history presented to the emergency department (ED) with visual hallucinations, limited ability to communicate, and the inability to move his right lower extremity. In addition, the patient experienced multiple apneic incidents in the ED. His delirium was determined to be secondary to taking a single 5-325 mg hydrocodone tablet. Administration of naloxone reversed the patient's symptoms.

**Conclusion:**

Cases reporting the development of delirium in patients who have taken opioid-based medications have been published, but these cases involved combinations of medications, higher doses of opioids than our patient ingested, and presentations after multiple days of drug use. Our case is unique given the patient's acute onset of symptoms 4 hours after hydrocodone ingestion, as well as the low dose that triggered his symptoms.

## INTRODUCTION

Opium, a latex compound derived from the *Papaver somniferum* plant, forms the basis of opioid-based medications. Many cultures had been using the plant for centuries for a variety of purposes by the time the sea-based opium trade developed in the 1700s.^1^ In the modern day, opioid-based medications are most frequently used for pain control. While the analgesic effects are well understood, individuals experience considerable variability in potency and the degree of pain relief.^2^ A standard dose of an opioid medication may lead to complete relief of symptoms for some patients but have little to no effect for others. The reasons for response variability are not fully understood and are likely to be multifactorial. Variability also occurs in side effects. Opioid-based medications are well known to cause a range of side effects including nausea, vomiting, constipation, dizziness, and respiratory depression.^3^ Among the less common side effects, psychosis and delirium have been documented. Psychosis is defined as the “presence of hallucinations (without insight into their pathologic nature), delusions, or both hallucinations without insight and delusions,”^4^ and delirium is defined as a “disturbance in attention and awareness.”^5^ Delirium is an uncommon side effect of opioids that has been rarely described in the literature.^6^ Typically, delirium is observed in older chronic users who are receiving end-of-life care and have regular daily opioid consumption.^6^ In rarer instances, however, delirium can also occur in younger and otherwise healthy patients.^7^ We present a case of a 20-year-old male with no prior psychiatric history who presented to the emergency department (ED) in acute delirium just hours after taking a single hydrocodone tablet.

## CASE REPORT

A 20-year-old male with a history of right inguinal hernia surgery was brought to the ED by a friend on postoperative day 1 secondary to acute onset paresthesias in his right lower extremity, inability to move this extremity, and altered mental status. The patient was 6 feet 4 inches tall, weighed 91 kilograms, had no noteworthy medical problems, and was a member of the football team at a local university. The patient's friend reported that the patient was recovering well postoperatively with mild pain that he managed conservatively with 600 mg ibuprofen. Before going to sleep, the patient took a single 5-325 mg hydrocodone tablet. Approximately 4 hours later, the patient's friend received a phone call from the patient stating he was in severe pain and could no longer move his right leg. The patient's friend noted that the patient was acting “strange” and was “incomprehensible” at times, prompting his decision to take the patient to the ED.

The patient's vital signs were stable. Physical examination showed a fresh surgical incision in the right inguinal region, erythematous discoloration in the right lower abdomen and testicle, tenderness to palpation of these areas, and an ON-Q (Avanos Medical, Inc) ropivacaine pump that had been placed during surgery the day prior.

From a neurologic standpoint, the patient was unable to move any part of his right lower extremity, including his right foot. He also subjectively endorsed paresthesias in the right lower extremity. The ability to take further history was limited as the patient's capacity to communicate was decreased, and at times he appeared to be responding to internal stimuli or was noncompliant during the interview. Initial differential diagnoses included cerebral vascular accident, pump malfunction, and a psychogenic etiology.

Complete blood count and comprehensive metabolic panel were unremarkable. During the next 2 hours, while awaiting computed tomography (CT) scan of his head, the patient had multiple apneic episodes, with oxygen saturations as low as 80%. In response, the patient was administered 0.4 mg of naloxone intravenously and shortly afterwards became alert, oriented, and able to communicate with ED staff. On physical examination, he was able to fully mobilize his right extremity, and according to his friend, had returned to his mental baseline. The patient endorsed amnesia and had no memory of being brought to the ED or of having any conversations while there. The CT was canceled and education about the importance of abstaining from opioid-based medications was provided. The patient was discharged in stable condition with plans to follow up with his surgeon in clinic.

## DISCUSSION

This case is noteworthy because of the acute onset of delirium, the relatively small dose of hydrocodone, and the young age of the patient. While the literature on delirium secondary to opioid use in otherwise healthy patients is limited, to our knowledge, no cases have reported such profound symptoms after a single dose. The limited cases typically involve contributing factors, such as high doses during an extended time period. For example, Tumenta et al described a patient who was on a home dose of hydromorphone (4 mg every 4 hours as needed) who developed psychosis while being treated for a sickle cell crisis.^8^ The patient received 8 mg of hydromorphone on admission and several subsequent doses. On the third day of hospitalization, the patient began exhibiting persecutory delusions.^8^

Our patient's side effect profile has been associated with opioid withdrawal. Casado-Espada et al reported the case of a 36-year-old male with no psychiatric history who took oxycodone consistently for chronic pain. After stopping the oxycodone acutely, the patient developed delusions and hallucinations along with “behavior and emotional changes” that eventually were shown to be secondary to withdrawal.^9^

Another consideration with our case was the patient's concurrent use of a nonsteroidal anti-inflammatory drug (NSAID). Our patient took 600 mg of ibuprofen 4 to 5 hours after his surgery. However, a minimum of 6 hours passed after the ibuprofen dose before he took the hydrocodone, and he did not show any signs of delirium during that time. NSAIDs have documented side effects, including NSAID-induced depression and paranoia.^10^ However, to our knowledge, no studies have examined the combination of NSAIDs and opioids as a cause of delirium. Research is required to determine if this combination could explain our patient's presentation.

Cases similar to our presentation have been reported, although with a slower onset of symptoms. Rasheed et al reported the case of 35-year-old male with no significant medical history who presented to the ED in acute psychosis and voicing suicidal ideation.^11^ The patient had been prescribed 7.5-325 hydrocodone 1 week prior and had been taking the medication every 4 to 6 hours as needed for lower back pain. He began developing symptoms 3 days prior to presentation. Blood work and CT of the head were unremarkable. The patient was admitted for observation, the hydrocodone was discontinued, and the patient's symptoms resolved within 2 days. At his 1-year follow-up with Psychiatry, the patient showed no signs of psychiatric instability.^11^

Because of the limited number of case reports available, identifying a definitive cause for the pathophysiology of our patient's presentation is challenging. Opioid-based medications can slow respiration and lead to apnea through the binding of mu-opioid receptors^12^ as in our patient; however, his acute delirium is less understood. One proposed theory relates to the antidopaminergic effects of opioids and their subsequent influence on increasing dopamine levels.^13^ Elevated dopamine levels can trigger downstream effects and lead to increased hypothalamic-pituitary-adrenal axis stimulation through the action of D1 and D2 receptors.^14^ Hypothalamic-pituitary-adrenal axis hyperactivity has been reported as a trigger for delirium.^15^ Genetic mutation is another proposed mechanism for opioid sensitivity. In a meta-analysis of studies reporting the impact of genetic variations on pain control, opioid consumption, and side effects, Ren et al identified multiple single-nucleotide polymorphisms that were associated with varying phenotypic responses to pain relief.^16^ While no study in the Ren et al analysis specifically addressed opioid-induced delirium,^16^ a single-nucleotide polymorphism could have contributed to our patient's presentation.

## CONCLUSION

Our case is unique because of the rapid onset of symptoms following a single low dose of hydrocodone. Despite his normal size and unremarkable medical history, our patient was uniquely sensitive to the medication, and his presentation had similarities to an overdose. Research is needed to understand the etiology of dramatic and unexpected responses to opioid-based medications. Before prescribing any opioids, clinicians must inquire whether patients have had adverse reactions to opioids (or any medications), because although rare, these side effects can be life-threatening. For patients taking opioid-based medications, symptoms such as altered mental status, changes in behavior, decreased ability to communicate, hallucinations, and apnea require proper treatment and reversal.

## Notes

*This article meets the Accreditation Council for Graduate Medical Education and the American Board of Medical Specialties Maintenance of Certification competencies for Patient Care and Medical Knowledge.*
